# A Robust Distributed Multipoint Fiber Optic Gas Sensor System Based on AGC Amplifier Structure

**DOI:** 10.3390/s16081187

**Published:** 2016-07-28

**Authors:** Cunguang Zhu, Rende Wang, Xuechen Tao, Guangwei Wang, Pengpeng Wang

**Affiliations:** School of Physics and Technology, University of Jinan, Jinan 250022, China; sps_zhucg@ujn.edu.cn (C.Z.); 15253135712@163.com (R.W.); 15562415086@163.com (X.T.); 15106910621@163.com (G.W.)

**Keywords:** gas sensor, AGC, distributed multipoint sensor, optical transmission loss

## Abstract

A harsh environment-oriented distributed multipoint fiber optic gas sensor system realized by automatic gain control (AGC) technology is proposed. To improve the photoelectric signal reliability, the electronic variable gain can be modified in real time by an AGC closed-loop feedback structure to compensate for optical transmission loss which is caused by the fiber bend loss or other reasons. The deviation of the system based on AGC structure is below 4.02% when photoelectric signal decays due to fiber bending loss for bending radius of 5 mm, which is 20 times lower than the ordinary differential system. In addition, the AGC circuit with the same electric parameters can keep the baseline intensity of signals in different channels of the distributed multipoint sensor system at the same level. This avoids repetitive calibrations and streamlines the installation process.

## 1. Introduction

One of the most important actual problems in the gas detection field is that there are strong demands for environmentally hazardous gas detection to prevent explosions or poisoning accidents [1,2,3]. Optical gas sensors play an important role in the sensing field for the measurement of combustible and explosive gas under harsh environmental conditions [4,5,6,7]. As a kind of optical sensing technology, tunable diode laser absorption spectroscopy (TDLAS) is widely used because of its advantages such as excellent insulation properties, high sensitivity, wide range, long life, etc. Moreover, matching with a fiber-optic network technology has added the prospect of broad application in the field of distributed multipoint detection [8,9,10,11].

Direct absorption spectroscopy is the simplest and most direct detection technique in TDLAS [12,13]. It often employs the self-balanced technology for demodulating absorption signals, which has been described in detail in previous research [14]. A distributed feedback laser diode (DFB-LD) emission scanning across a gas absorption wavelength is split into two beams by a 1 × 2 fiber coupler. One is directly coupled on a photoelectric detector (PD), which serves as the reference signal input of the self-balanced circuit. The other beam is coupled on a similar PD after being passed through the gas cell, which is received by the second input of the self-balanced circuit. Then, the absorption peak can be obtained by self-balanced demodulation such as a differential or balanced ratiometric detector (BRD) circuit. Intuitive and original absorption line shapes can be used to determine the gas properties [15,16].

However, besides gas absorption, there could be many other factors such as temperature change, fiber bending loss and the laser aging process, which can cause the transmission intensity to drop in real-world applications [17,18]. By contrast, the variation of light intensity induced by these factors can be more obvious. Undoubtedly, this is a principal reason for accuracy deterioration of TDLAS sensor system. Two inputs of the self-balanced demodulation circuit in direct absorption spectroscopy require a strict balance to be met in order to obtain the absorption spectrum with a horizontal baseline. Otherwise, the baseline tilt will lead to a serious distortion of the absorption line, which could result in further obvious measurement errors [19].

In this paper, a robust distributed multipoint fiber optic gas sensor system based on an automatic gain control (AGC) amplifier structure is proposed. The AGC amplifier adopts its internal self-gain amplification function for adjustment and stabilization of the baselines of the two inputs of the self-balanced demodulation circuit to the same magnitude. In this way, not only can the attenuation of the signal caused by the transmission loss be effectively compensated, but also a multi-point simultaneous detection can be easily realized without the affection of insertion loss differences of gas cells in different detection channels.

## 2. Theory of AGC Amplifier

### 2.1. Beer-Lambert Law

The measurement technique is based on the absorption of monochromatic near-IR radiation. The transmission of the probe beam through a uniform absorbing medium follows the Beer-Lambert law:

(1) $$I\left(\upsilon \right)\;=\;I_{0}\left(\upsilon \right)\text{exp}\left[-\alpha \left(\upsilon \right)CL\right]$$

where *C* is the target gas concentration, and *L* is the length of the absorption path. The concentration *C* can be obtained by identifying the emergent light intensity *I*(*υ*) and the incident light intensity *I*_0_(*υ*), α(*υ*) represents the absorption coefficient at the light wave number *υ*, it can be expressed as:

(2) α(υ)=PS(T)ϕ(υ)

where *P*(Pa) is the total pressure, *S*(*T*) (cm∙mol^−1^) is the line strength at an arbitrary temperature (*T*), *ϕ*(*υ*) (cm) is the linear function of absorption spectra.

### 2.2. Fiber Optic Gas Sensor System based on AGC Amplifier Structure

**Figure 1:**
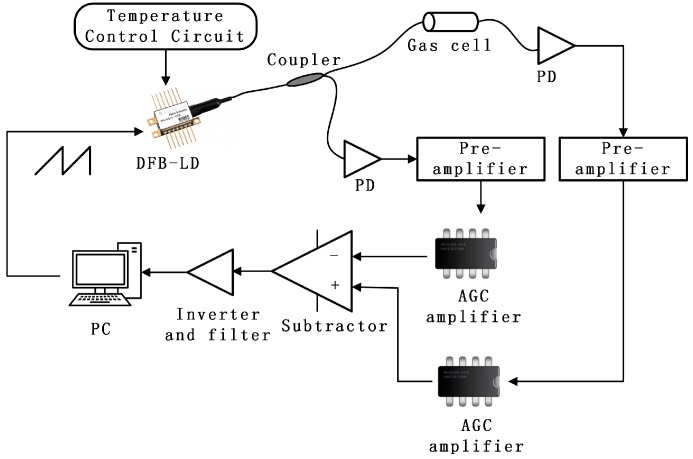
Schematic of the automatic gain control (AGC) amplifier detection system.

A block diagram of the fiber optic gas sensor system based on AGC amplifier structure is shown in Figure 1. An AGC module is added between the pre-amplifier and the self-balanced circuit (differential circuit). The baseline amplitudes of the detection signal and the reference signal before the differential circuit can be amplified to the same level through the AGC circuit no matter how the transmission loss changes.

The intensities of the two signals (the reference signal *I*_ref_(*υ*) and the detection signal *I*_det_(*υ*)) coupled into the AGC circuits are described below:

(3) $$I_{\text{ref}}\left(\upsilon \right)\;=\;\gamma 1\left(t\right)I_{0}\left(\upsilon \right)$$

(4) $$I_{\text{det}}\left(\upsilon \right)\;=\;\frac{\gamma 2\left(t\right)}{\varepsilon }I_{0}\left(\upsilon \right)\text{exp}\left[-\alpha \left(\upsilon \right)CL\right]$$

where and *γ*2(*t*) is the time-varying optical transmission loss coefficient of the two beams due to various non-absorption losses, and *ε* is the coupler splitting ratio. The AGC circuit can achieve a timely gain adjustment to maintain a stable baseline according to the reset thresholds even if the *γ*1(*t*) and *γ*2(*t*) are continually changing. The two signal outputs by the AGC circuits are given by:

(5) $$\frac{1}{\gamma 1\left(t\right)}I_{\text{ref}}\left(\upsilon \right)\;=\;I_{0}\left(\upsilon \right)$$

(6) $$\frac{\varepsilon }{\gamma 2\left(t\right)}I_{\text{det}}\left(\upsilon \right)=I_{0}\left(\upsilon \right)\text{exp}\left[-\alpha \left(\upsilon \right)CL\right]$$

The final signal processed by the differential circuit is given by:

(7) $$I_{\text{out}}\;=\;\frac{1}{\gamma 1\left(t\right)}I_{\text{ref}}\left(\upsilon \right)\;-\;\frac{\varepsilon }{\gamma 2\left(t\right)}I_{\text{det}}\left(\upsilon \right)\;=\;I_{0}\left(\upsilon \right)\;-\;I_{0}\left(\upsilon \right)\text{exp}\left[-\alpha \left(\upsilon \right)CL\right]$$

When exp [−α(*υ*)*CL*] is much less than 1,

(8) exp[−α(υ)CL] ≈ 1−α(υ)CL

When *I*_0_(*υ*) did not change, the final signal (*I*_out_(*υ*)) will be proportional to the concentration of the gas to be measured, so the gas concentration (*C*) can be derived from the absorption peak.

### 2.3. AGC Amplifier

The AGC amplifier is an automatic amplification gain adjustment device. Using the AGC amplifier can guarantee a relatively stable output in the case of an unstable input.

**Figure 2:**
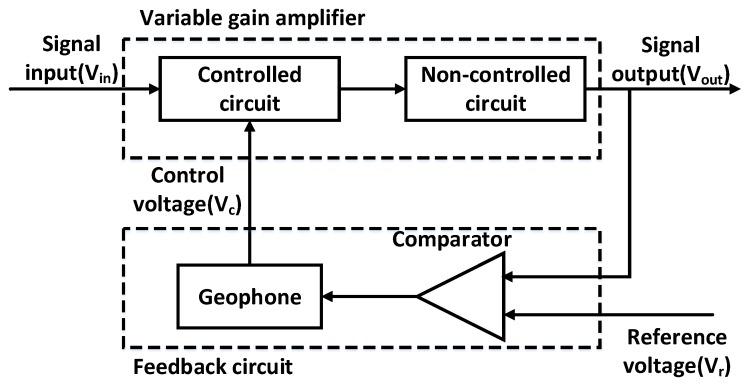
AGC amplifier structure diagram.

A closed loop AGC amplifier as shown in Figure 2 is used in the system. It is actually a negative feedback system, which is composed of a variable gain amplifier and a feedback circuit. The variable gain amplifier contains a controlled and a non-controlled amplifier circuit unit. A comparator and a detection circuit form a feedback circuit. The effect of the feedback circuit is to convert the output signal (V_out_) into a direct current (DC) voltage to achieve gain control (Comparator and detection circuit).

The input signal (V_in_) is time-variable due to transmission loss and other interferences, which is introduced by the input port of the controlled circuit unit and finally output by the non-controlled circuit unit. The V_out_ can remain steady and be fed to the next stage circuit (differential circuit). 

When V_in_ is small, V_out_ is also smaller than the reference voltage (V_r_). The output of the feedback circuit is zero. Thus, gain regulation does not occur. The system maintains full-scale amplification. 

When the input signal reaches a certain value (V_1_ < V_in_ < V_2_), and as a result thus V_out_ > V_r_, the difference between V_out_ and V_r_ can be served as the input of the feedback circuit. At this point, the gain control voltage (V_c_) of the controlled circuit unit will change and result in a gain adjustment to keep the V_out_ in the vicinity of the V_r_.

**Figure 3:**
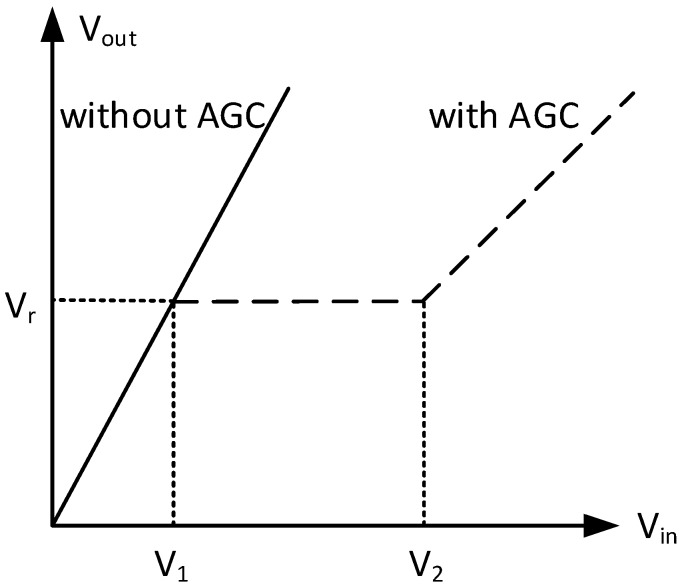
Static regulation characteristic of AGC amplifier. Among them, V_1_ is the threshold voltage, and the V_2_ is out of control voltage, they are two key performance indicators of the AGC amplifier.

When V_in_ is large (V_in_ > V_2_), AGC amplifier no longer works, the system is amplified with a minimum gain, as shown in Figure 3.

## 3. Experimental Apparatus and Results

Stress-induced fiber bending will inevitably occur and cause a transmission loss in the long-haul optical fiber transmission. In a multi-point detection system, the amount of loss between different monitoring points must be distinct and unsynchronized. This will affect the TDLAS detection system far more than some random high frequency noise.

A fiber bending test is used to evaluate the reliability of the system. Acetylene is selected as the gas to be detected. A single-mode DFB-LD (WSLS-137010C1424-20, Agilecom, Fiber Solution, San Jose, CA, USA) is used as the light source, in which the output wavelength is modulated by adjusting the laser temperature and the injection current. The injection current is scanned with a saw-tooth function, which ensures that the light emitted from the laser source can cover the entire wavelength range of acetylene absorption lines near 1532 nm. Laser temperature is set to a suitable working point by a micro-temperature controlling chip (LTC 1923, Linear Technology, Milpitas, CA, USA). The LD beam is split into reference and detection beams by a 1 × 2 coupler.

**Figure 4:**
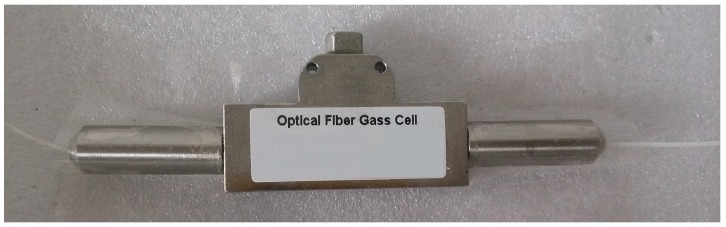
The gas cell structure.

The detection beam passes through a gas cell consisting of a stainless-steel tube. The gas cell shown in Figure 4 is filled with 1000 ppm acetylene in which the absorption path is 3 cm. A good air seal structure ensures that we do not need to consider the measurement errors caused by an unstable air concentration.

**Figure 5:**
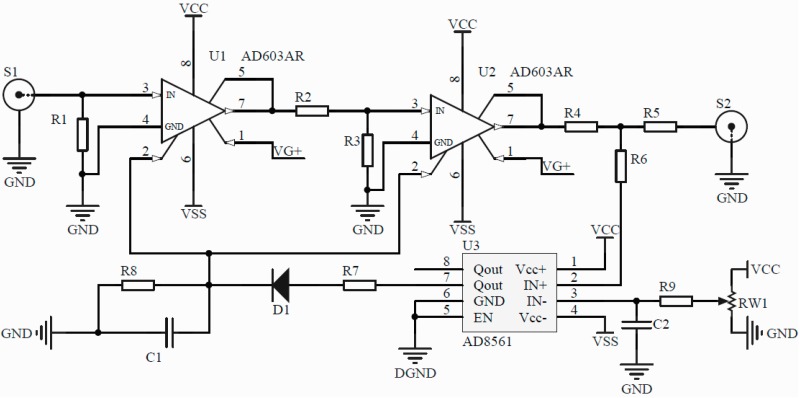
The circuit diagram of the AGC amplifier.

An ordinary differential demodulation system is employed to compare with the AGC differential demodulation system in the test. They choose the same pre-amplifier and differential circuit. The only difference is whether to join the AGC module. In the AGC system, the controlled circuit unit is constructed with the AD603 chip as shown in Figure 5.

**Figure 6:**
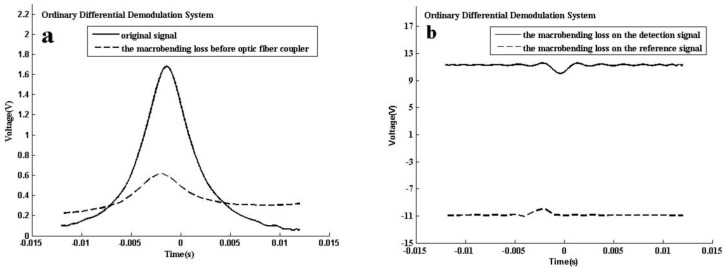

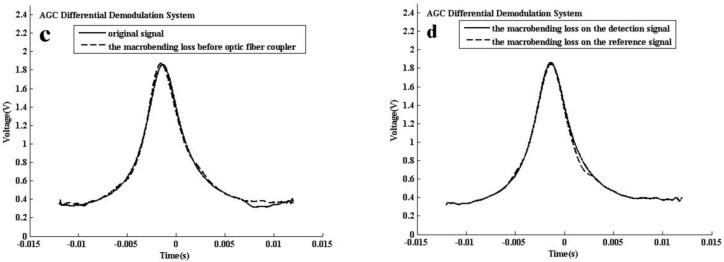
Macro bending loss test results of the different systems. (**a**) The change of the absorption waveform obtained by the ordinary differential demodulation system under fiber bending before the coupler; (**b**) The change of the absorption waveform obtained by the ordinary differential demodulation system under fiber bending on the detection signal or the reference signal; (**c**) The change of the absorption waveform obtained by the AGC differential demodulation system under fiber bending before the splitter; (**d**) The change of the absorption waveform obtained by the AGC differential demodulation system under fiber bending on the detection signal or the reference signal.

In the ordinary system, the baseline of the two signals (the detection signal and the reference signal) can achieve the same magnitude by adjusting the preamplifier. The amplitude of the final demodulated signal is 1.668 V. Bending with a bending radii of 5 mm causes propagation losses in an optical fiber between the DFB-LD and coupler, and magnitude of the demodulated signal changes to 0.321 V. The measurement error is 80.76%. As shown in Figure 6a, although there is a large amplitude attenuation, a relatively significant absorption profile can be still detected because the amounts of light loss of the two signals are the almost same. However, the macro bending loss occurs on the transmission fiber of the detection signal or on the transmission fiber of the reference signal. This situation is especially serious in profile measurement as shown in Figure 6b.

We have replicated this experiment on the AGC differential demodulation system. Without a fiber bending loss, the magnitude of the demodulated signal is 1.527 V. As shown in Figure 6c, when a 5 mm macro bending loss occurs before the fiber coupler, the demodulated signal changes to 1.491 V. The measurement error is reduced to 2.36%. As shown in Figure 6d, when the macro bending loss occurs on the transmission fiber of the reference signal or on the detection signal, the measurement errors are 3.80% (1.527–1.469 V) and 4.06% (1.527–1.465 V), respectively. Reliability of the system has been enhanced.

**Figure 7:**
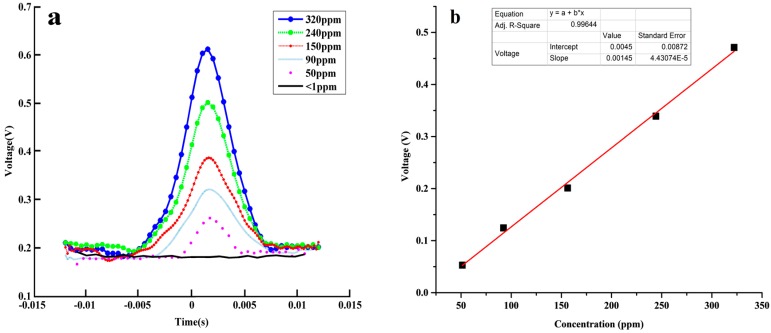
(**a**) Absorption line shapes of acetylene 1532.83 nm line at different concentration; (**b**) Peak absorbance of demodulated signal as a function of acetylene concentration in the gas cell.

Acetylene concentration measurement in different mixtures of acetylene and nitrogen is performed. Absorption line shape under different acetylene concentrations in a 20 cm gas cell is shown in Figure 7a. As shown in Figure 7b, the demodulated signal is well proportional to acetylene concentration ranging from <1 ppm to 320 ppm. The proportional relationship is described by a linear equation with an R-square of 0.99644. The detected noise suppressing based on a 256-time average is lower than 1.30 mV in practical application. According to the analysis of slope 1.45 mV/ppm of the linear equation, the deduced minimum absorption sensitivity could be improved to 2 × 10^−5^, which means the minimum concentration for acetylene at 1532.83 nm would be 896.6 ppb for just a 20-cm path length.

**Figure 8:**
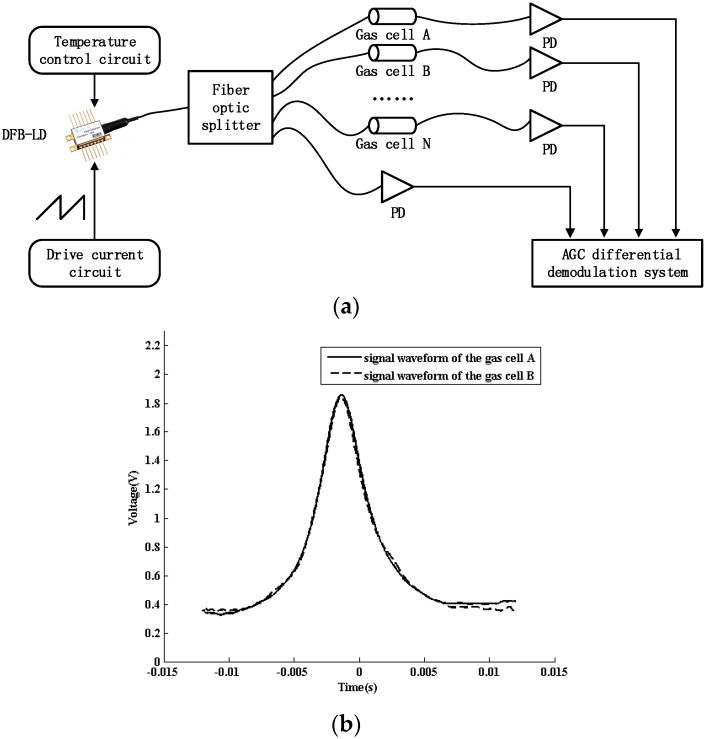
(**a**) The AGC differential demodulation system with a fiber optic splitter for multipoint detection; (**b**) Comparison of signal waveforms with different gas cells.

A fiber optic splitter replacing the 1 × 2 fiber coupler is connected to several gas cells with different insertion losses to simulate multi-point detection, as shown in Figure 8a. If circuit parameters of the AGC module are fixed, undistorted spectroscopy can be demodulated from each channel without artificial regulation. As shown in Figure 8b, the amplitudes of absorption peaks after demodulation are basically the same on the premise that gas cells are aerated with acetylene at the same concentration.

## 4. Conclusions

A harsh environment-oriented multipoint fiber optic gas sensor system realized by AGC technology is proposed. Compared to the ordinary differential demodulation system, an AGC module is added between the pre-amplifier and the self-balanced circuit (differential circuit). To improve the photoelectric signal reliability, the electronic variable gain can be modified in real time by an AGC closed-loop feedback structure to compensate for optical transmission loss which is caused by the fiber bend loss or other reasons. The system reduces the signal attenuation caused by all kinds of loss, and improves the reliability of the detection. The deviation of the system based on AGC structure is below 4.02% when photoelectric signal decays due to fiber bending loss for a bending radius of 5 mm, which is 20 times lower than the ordinary differential system. In addition, an AGC circuit with same electric parameters can keep the baseline intensity of signals in different channels of the multiplexing sensor system at the same level. This avoids repetitive calibrations and simplifies the installation process.

## References

[1] Wetchakun K., Samerjai T., Tamaekong N., Liewhiran C., Siriwong C., Kruefu V., Wisitsoraatb A., Tuantranontb A., Phanichphant S. (2011). Semiconducting metal oxides as sensors for environmentally hazardous gases. Sens. Actuator B Chem..

[2] Hawe E., Fitzpatrick C., Chambers P., Dooly G., Lewis E. (2008). Hazardous gas detection using an integrating sphere as a multipass gas absorption cell. Sens. Actuator A Phys..

[3] Tan Q., Pei X., Zhu S., Dong S., Liu J., Xue C., Liang T., Zhang W., Xiong J. (2013). Development of an optical gas leak sensor for detecting ethylene, dimethyl ether and methane. Sensors.

[4] Levitsky I.A. (2015). Porous silicon structures as optical gas sensors. Sensors.

[5] Bae M.K., Lim J.A., Kim S., Song Y.W. (2013). Ultra-highly sensitive optical gas sensors based on chemomechanical polymer-incorporated fiber interferometer. Opt. Express.

[6] Pickrell G., Peng W., Wang A. (2004). Random-hole optical fiber evanescent-wave gas sensing. Opt. Lett..

[7] Miehael E.W., Suhong K., Scott T.S. (2001). In-situ combustion measurements of CO_2_ by use of a distributed feedbaek diode laser sensor near 2.0 μm. Appl. Opt..

[8] Hunsmann S., Wunderle K., Wagner S., Rascher U., Schurr U., Ebert V. (2008). Absolute, high resolution water transpiration rat measurements on single plant leaves via tunable diode laser absorption spectroscopy (TDLAS) at 1.37 μm. Appl. Phys. B.

[9] Linnerud I., Kaspersen P., Jæger T. (1998). Gas monitoring in the process industry using diode laser spectroscopy. Appl. Phys. B.

[10] Zhang S., Wang Q., Zhang Y., Song F., Chen K., Chou G., Chang J., Wang P., Kong D., Wang Z. (2012). Water vapor detection system based on scanning spectra. Photonic Sens..

[11] Duffin K., McGettrick A.J., Johnstone W., Stewart G., Moodie D.G. (2007). Tunable diode laser spectroscopy with wavelength modulation: A calibration-free approach to the recovery of absolute gas absorption line-shapes. IEEE J. Lightw. Technol..

[12] Durry G., Megie G. (2000). In situ measurements of H_2_O from a stratospheric balloon by diode laser direct-differential absorption spectroscopy at 1.39 microm. Appl. Opt..

[13] Berrou A., Raybaut M., Godard A., Lefebvre M. (2010). High-resolution photoacoustic and direct absorption spectroscopy of main greenhouse gases by use of a pulsed entangled cavity doubly resonant opo. Appl. Phys. B.

[14] Zhu C.G., Chang J., Wang P.P., Wang Q., Wei W., Liu Z., Zhang S.S. (2014). Single-beam water vapor detection system with automatic photoelectric conversion gain control. Opt. Commun..

[15] Zhu C.G., Chang J., Wang P.P., Sun B.N., Wang Q., Wei W., Liu X.Z., Zhang S.S. (2014). Improvement of measurement accuracy of infrared moisture meter by considering the impact of moisture inside optical components. IEEE Sens. J..

[16] Ono S., Wang D.T., Gruen D.S., Lollar B.S., Zahniser M.S., Mcmanus B.J., Nelson D.D. (2014). Measurement of a doubly substituted methane isotopologue, ^13^CH_3_D, by tunable infrared laser direct absorption spectroscopy. Anal. Chem..

[17] Zhu C.G., Chang J., Wang P.P., Wang W.J., Wang Q., Liu Y.N., Lv G., Liu X.Z., Wei W., Wang F.P. (2013). Reliability analysis and comparison of demodulation methods for dual-beam wavelength-modulation spectroscopy water vapor detection. Appl. Opt..

[18] Wang Q., Farrell G., Freir T. (2005). Theoretical and experimental investigations of macro-bend losses for standard single mode fibers. Opt. Express.

[19] Arroyo M.P., Hanson R.K. (1993). Absorption measurements of water-vapor concentration, temperature, and line-shape parameters using a tunable InGaAsP diode laser. Appl. Opt..

